# Silicon Nanowire-Assisted High Uniform Arrayed Waveguide Grating

**DOI:** 10.3390/nano13010182

**Published:** 2022-12-30

**Authors:** Shuo Yuan, Jijun Feng, Zhiheng Yu, Jian Chen, Haipeng Liu, Yishu Chen, Song Guo, Fengli Huang, Ryoichi Akimoto, Heping Zeng

**Affiliations:** 1 Shanghai Key Laboratory of Modern Optical System, Engineering Research Center of Optical Instrument and System, Ministry of Education, School of Optical-Electrical and Computer Engineering, University of Shanghai for Science and Technology, Shanghai 200093, China; 2 Key Laboratory of Medical Electronics and Digital Health of Zhejiang Province, Jiaxing University, Jiaxing 314001, China; 3 Research Institute for Advanced Electronics and Photonics, National Institute of Advanced Industrial Science and Technology, Tsukuba 305-8568, Japan; 4 Chongqing Key Laboratory of Precision Optics, Chongqing Institute of East China Normal University, Chongqing 401120, China; 5 State Key Laboratory of Precision Spectroscopy, East China Normal University, Shanghai 200241, China

**Keywords:** arrayed waveguide grating, nanowire, uniformity, silicon photonics

## Abstract

Determining how to improve the non-uniformity of arrayed waveguide grating (AWG) is of great significance for dense wavelength division multiplexing (DWDM) systems. In this work, a silicon nanowire-assisted AWG structure is proposed, which can achieve high uniformity with a low insertion loss. The article compares the effect of nanowire number and shape on uniformity and insertion loss, finding that double nanowires provide the best performance. Double nanowires with a width of 230 nm and length of 3.5 μm can consist of a slot configuration between arrayed waveguides, both connecting to the star coupler and spacing 165 nm from the waveguides. Compared with conventional 8- and 16-channel AWGs with channel spacing of 200 GHz, the non-uniformity of the presented structure can be improved from 1.09 and 1.6 dB to 0.24 and 0.63 dB, respectively. The overall footprint of the device would remain identical, which is 276 × 299 or 258 × 303 μm^2^ for the 8- or 16-channel AWG. The present high uniformity design is simple and easy to fabricate without any additional insertion loss, which is expected to be widely applied in the highly integrated DWDM systems.

## 1. Introduction

High-speed and broadband communication systems play important roles for daily life [1,2]. Dense wavelength division multiplexing (DWDM) technology has attracted much attention due to its ability to increase communication capacity easily [3,4]. Among different DWDM technologies, arrayed waveguide grating (AWG) is one of the most commonly used technical routes, due to its small crosstalk, low loss, and compact integration [5,6,7]. AWGs have been demonstrated in some low-refractive-index-contrast materials [8,9], such as InP [10], silica [11], and polymer [12]. However, all these materials have large footprints and bending losses [13,14]. Owing to the high refractive-index-contrast, silicon-based AWGs can be made very compact [15,16,17,18], which also allows for low-cost, high-volume manufacture due to their complementary metal–oxide semiconductor (CMOS) compatible processing [19,20].

However, the non-uniformity for traditional silicon-based AWGs still needs to be improved. The insertion loss of the edge channel would be about 3 dB higher than those of the center ones when free spectral range (FSR) is fully utilized [13,21,22]. Additional light power needs to be added to maintain the same bit error rate, ultimately affecting the power budget of the entire communication system [22,23,24,25]. After long-distance transmission, the signal-to-noise ratio of the traditional AWG would decrease seriously [26]. To solve this issue, many methods have been proposed such as mode field converters [27,28,29], optical combiner structures [30], and slab waveguide configurations [31]. Mode field converters usually require careful design and fine processing. Optical combiner structures increase the overall size as well as additional insertion loss. Slab waveguide configurations require additional transition area between ridge and slab structure which would result in excess losses. Moreover, some special designs at the interface of the arrayed waveguides and the free-propagation region were proposed. For example, a cyclic 16-channel AWGR shows a non-uniformity of approximately 1.1 dB and an additional insertion loss of 2.3 dB [32]. A 12-channel AWG using multimode interference couplers can achieves a non-uniformity of 0.8 dB but greatly increases the device size [33]. Assisted waveguides have been proposed recently, which could maintain the device size and insertion loss [34]. However, it could not solve the channel non-uniformity well, and the design still needs further optimization.

In this work, a silicon nanowire-assisted AWG is proposed, which can achieve high uniformity with a low insertion loss. The article compares the effect of nanowire number and shape on uniformity and insertion loss, finding that double nanowires provide the best performance. Double nanowires are used here to consist of a slot configuration between the arrayed waveguides and connect to the star coupler, which makes it different from the traditional design. Compared with conventional 8- and 16-channel AWGs with channel spacing of 200 GHz, the non-uniformity of the presented structure can be improved from 1.09 and 1.6 dB to 0.24 and 0.63 dB, respectively. Little change happens for the device’s overall size by the introduction of the nanowires into the gaps between the arrayed waveguides. The overall footprint of the device would remain identical to the conventional design, which is 276 × 299 or 258 × 303 μm^2^ for the 8 or 16-channel AWG. Moreover, the present simple design has no additional insertion loss. Additionally, a commercially available CMOS-compatible manufacturing equipment can be used for device fabrication. Thus, high-volume and low-cost production can be expected.

## 2. Device Structure and Design

**Figure 1:**
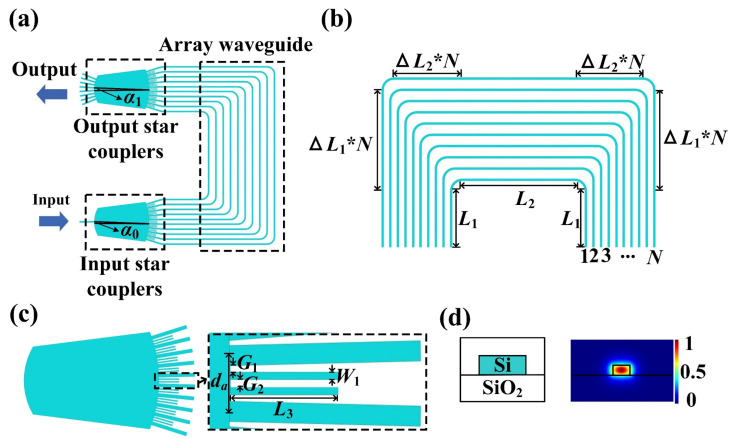
(**a**) Schematic of the nanowire-assisted AWG structure with (**b**,**c**) for the magnified view of arrayed waveguides and star coupler, respectively. (**d**) Waveguide platform and fundamental TE mode.

Figure 1a shows the schematic diagram of the proposed nanowire-assisted AWG. The beam diverges at the input star coupler, then propagates through the arrayed waveguide, and finally converges on the image plane of the output star coupler. Figure 1b,c show the detailed diagrams of the arrayed waveguides and star coupler, with the length difference between two adjacent arrayed waveguides of 2(∆*L*_1_ + ∆*L*_2_). Here, *L*_1_ and *L*_2_ are 39 and 120 μm, as well as the bending radius is 20 μm. As shown in Figure 1c, double nanowires with a width *W*_1_ of 230 nm and length *L*_3_ of 3.5 μm can consist of a slot configuration between arrayed waveguides, both connecting to the star coupler and spacing *G*_1_ of 165 nm from the arrayed waveguides. The spacing *G*_2_ between the double nanowires is 110 nm. The designed structure is based on a silicon-on-insulator (SOI) platform, with a 3-μm-thick buffering layer and a 220-nm-thick silicon core layer, as shown in Figure 1d. The silicon waveguide with a width *W* of 500 nm is employed, ensuring a single fundamental TE mode operation.

For the AWG design, there is a constant length difference between adjacent waveguides, which should equal an integer multiple of the central wavelength. The beam in each arrayed waveguide with the same wavelength arrives at the output star coupler with the same phase, and the light field distribution of the input star coupler will be reproduced in the output star coupler. As a result, the diverging beams in the input star coupler will converge into beams with the same amplitude and phase distribution on the image plane of the output star coupler. Due to the effect of waveguide dispersion, the focus point of the converged beam will move along the image plane of the output star coupler as the wavelength varies. Thus, the spatial separation of different wavelengths can be achieved by placing the output waveguides at an appropriate position on the image plane of the output star coupler [35].

For this AWG, the grating equation can be expressed as

*n_s_d_a_sinα_0_* + *n_a_*∆*L* + *n_s_d_a_sinα_1_* = *mλ,* (1)

where *n_s_* and *n_a_* are effective refractive indices of the star coupler and arrayed waveguides, *d_a_* is the space between adjacent arrayed waveguides on the tangent line, *α*_0_ and *α*_1_ are the input and output angles, ∆*L* = 2(∆*L*_1_ + ∆*L*_2_) is the length difference between adjacent arrayed waveguides, *m* is an integer diffraction order, and *λ* is the wavelength of the beam within the waveguides [36].

For the conventional AWG, the light field distribution at the arrayed waveguides approximates a Gaussian distribution, resulting in a Gaussian envelope-distributed beam focused on the image plane of the output star coupler. Thus, this would lead to a non-uniform light intensity distribution between the central and edge channels, which could be described by a non-uniformity *L_u_* defined as

*L_u_* = −10*lg*(*I_e_*/*I_c_*), (2)

where *I_e_* and *I_c_* are the light intensities in the edge and center channels, respectively [21]. By introducing nanowires at the array waveguide, the field distribution at the end of the waveguide can be disturbed. Furthermore, the perturbation of the field distribution on the image plane can be calculated using Kirchhoff–Huygens formula. The parameters of nanowires are adjusted constantly so that the flat light field distribution on the image plane can be obtained. Therefore, the power difference of the output channel placed on the image plane is reduced, and the non-uniformity can be suppressed. Here, 8- and 16-channel AWGs with improved non-uniformity are presented. Based on the impact of nanowires on non-uniformity, 8- and 16-channel AWGs with improved non-uniformity are designed and the main parameters are presented in Table 1.

## 3. Device Performance and Discussion

**Figure 2:**
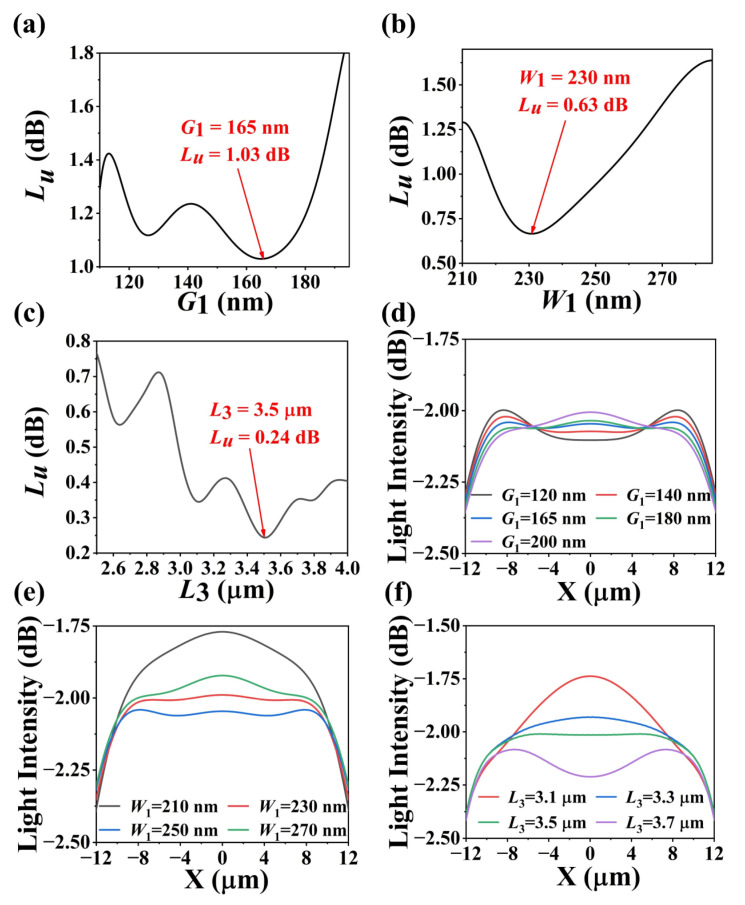
Simulated non-uniformity with the variation of (**a**) *G*_1_, (**b**) *W*_1_ and (**c**) *L*_3_. Light intensity distribution along the image plane of AWG with different values of (**d**) *G*_1_, (**e**) *W*_1_ and (**f**) *L*_3_.

In order to simulate the performance of the nanowire-assisted high uniform AWG, 2.5D-FDTD (Lumerical FDTD Solutions of 8.9.1584) method was used [37]. Perfectly matched layers (PML) were used to simulate boundary conditions. The mesh size of the simulation area was set to ∆*x* = ∆*y* = 50 nm and ∆*z* = 20 nm. The refractive indices were 1.444 and 3.476 for SiO_2_ and Si, respectively. When the wavelength was 1556 nm, the dispersion was about 1.4227 × 10^3^ ps/nm/km. The light source was set to TE mode with a center wavelength of 1556 nm. Additionally, the simulation areas of the 8 or 16-channel AWG were 310 × 290 or 450 × 350 μm^2^. Light intensity field distribution along the image plane of AWG with different values of *G*_1_, *W*_1_ and *L*_3_ were compared with the other two parameters unchanged as in Figure 2d–f. As shown in Figure 2d, it turns out that the ripple of light intensity distribution became the flattest at a *G*_1_ of 165 nm (blue line), while Figure 2e clearly shows that the flattest light intensity could be obtained at a *W*_1_ of 230 nm (red line). Additionally, Figure 2f indicates how the light intensity distribution was affected by nanowire length *L*_3_ and the flattest light intensity could be obtained at a *L*_3_ of 3.5 μm (green line).

**Figure 3:**
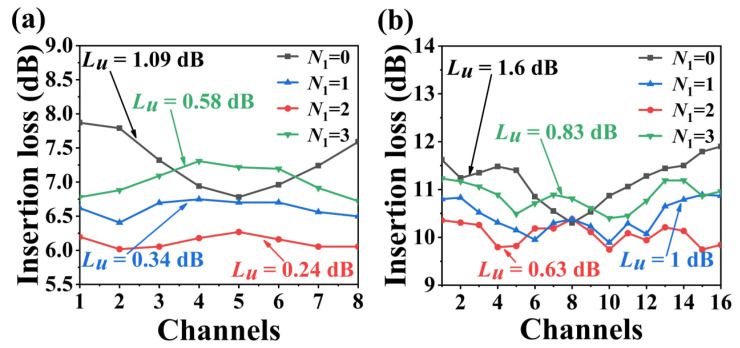
Simulated non-uniformity and insertion loss for all output channels of (**a**) 8 and (**b**) 16-channel nanowire-assisted AWG with the variation of *N*_1_.

It should be emphasized that double nanowires configuration is optimum for the non-uniformity improvement. Tapered nanowire may not help to improve the insertion loss and non-uniformity but greatly increase the complexity of the design [21]. Figure 3 shows the simulated non-uniformity and insertion loss for all output channels with the variation of nanowire number *N*_1_. The optimal parameters of nanowires vary with the nanowire number *N*_1_. When *N*_1_ is 1, the optimal parameters of nanowires are *G*_1_ = 110 nm, *W*_1_ = 440 nm and *L*_3_ = 5 μm. When *N*_1_ is 2, the optimal parameters of nanowires are *G*_1_ = 165 nm, *W*_1_ = 230 nm and *L*_3_ = 3.5 μm. When *N*_1_ is 3, the optimal parameters of nanowires are *G*_1_ = 140 nm, *W*_1_ = 160 nm and *L*_3_ = 3.4 μm. For both, the non-uniformity decreases first and then increases with the nanowire number *N*_1_. When *N*_1_ is 2, the channel’s non-uniformity can be minimized to 0.24 and 0.63 dB with a minimum insertion loss for the 8- and 16-channel AWGs. The additional nanowires can improve the coupling efficiency of arrayed waveguides and star couplers, reducing insertion loss. Too few nanowires can not make the light intensity flat, while too many ones will deteriorate the performance.

**Figure 4:**
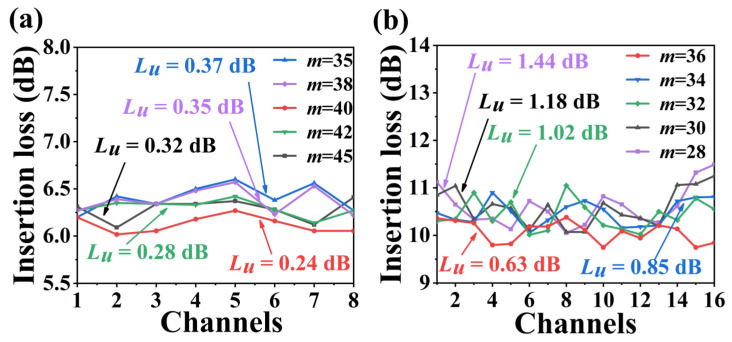
Simulated non-uniformity and insertion loss for all output channels of (**a**) 8 and (**b**) 16-channel nanowire-assisted AWG with the variation of the diffraction order *m*.

It is important to investigate the influence of diffraction order (*m*) on non-uniformity and insertion loss. Figure 4 demonstrates the non-uniformity and insertion loss of all output channels with change of diffraction order. The non-uniformity for an 8-channel AWG initially decreases and then increases when the diffraction order increases gradually from 35 to 45, as shown in Figure 4a. When *m* is 40, the non-uniformity can be minimized to 0.24 dB. For a 16-channel AWG, the non-uniformity gradually reduces as the diffraction order increases from 28 to 36 as in Figure 4b. The free spectral range of 25.99 nm in this AWG can be fully utilized at *m* = 36, and the minimum non-uniformity may be achieved at 0.63 dB.

**Figure 5:**
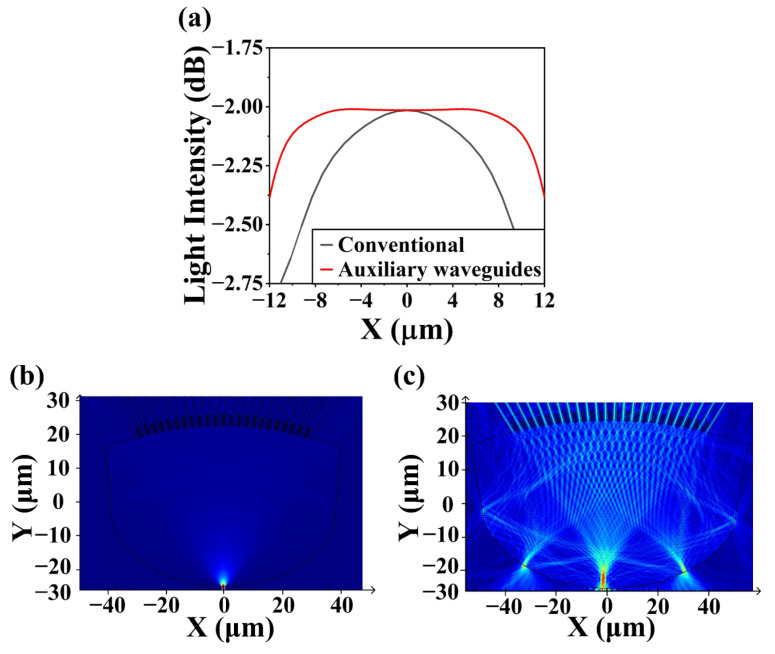
(**a**) Light intensity distribution along the image plane with conventional design and uniformity-improved design. Calculated electric field distribution of (**b**) the input star coupler and (**c**) the output star coupler.

Figure 5a shows the comparison of the light intensity distribution on the image plane between the conventional and optimized designs. By introducing nanowires between the arrayed waveguides, the variation of light intensity on the image plane of the output star coupler can be reduced, which ensures that each output waveguide can be obtained the same optical power and the non-uniformity can be dramatically reduced. As shown in Figure 5b, the calculated electric field distribution shows that the beam diverges in the input star coupler, then enters the arrayed waveguides homogeneously. In Figure 5c, the beam from the end of arrayed waveguides can pass through the output star coupler and converge on the image plane at a wavelength of 1556 nm.

**Figure 6:**
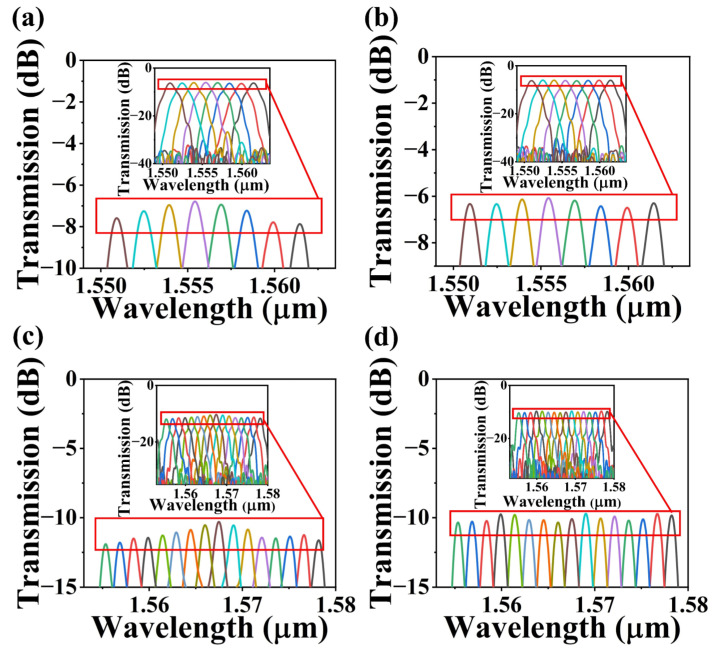
Simulated partial transmission spectra with the conventional and the uniformity-improved structure for (**a**,**b**) 8 and (**c**,**d**) 16-channel AWG. Inset: Simulated complete transmission spectra.

Figure 6 shows the spectral response of the 8- and 16-channel AWGs with the conventional and the nanowire-assisted design, respectively. The double nanowires with *G*_1_ = 165 nm, *W*_1_ = 230 nm and *L*_3_ = 3.5 μm can be chosen as the best parameters for subsequent simulations. For the 8-channel AWG as in Figure 6a,b, the non-uniformity is reduced from 1.09 to 0.24 dB as the insertion loss of the center channel is reduced from 6.78 to 6.26 dB. Meanwhile, the non-uniformity of the 16-channel AWG is reduced from 1.6 to 0.63 dB and the insertion loss of the center channel is reduced from 10.58 to 10.1 dB as in Figure 6c,d. The coupler loss between the waveguide and the star coupler and furthermore the excitation loss of the adjacent grating make up the majority of the insertion loss of the AWG. For 8- and 16-channel AWG, the excitation loss of adjacent gratings are 3.18 and 3.95 dB, respectively, and the coupling loss are 3.08 and 6.15 dB. The AWG has little scattering and absorption loss. When the bending radius of the waveguide is greater than 5 μm, the bending loss is negligible [38]. Hence, the nanowire-assisted AWG can greatly improve the non-uniformity of the channel and reduce the insertion loss, which is beneficial for the development of WDM systems.

**Figure 7:**
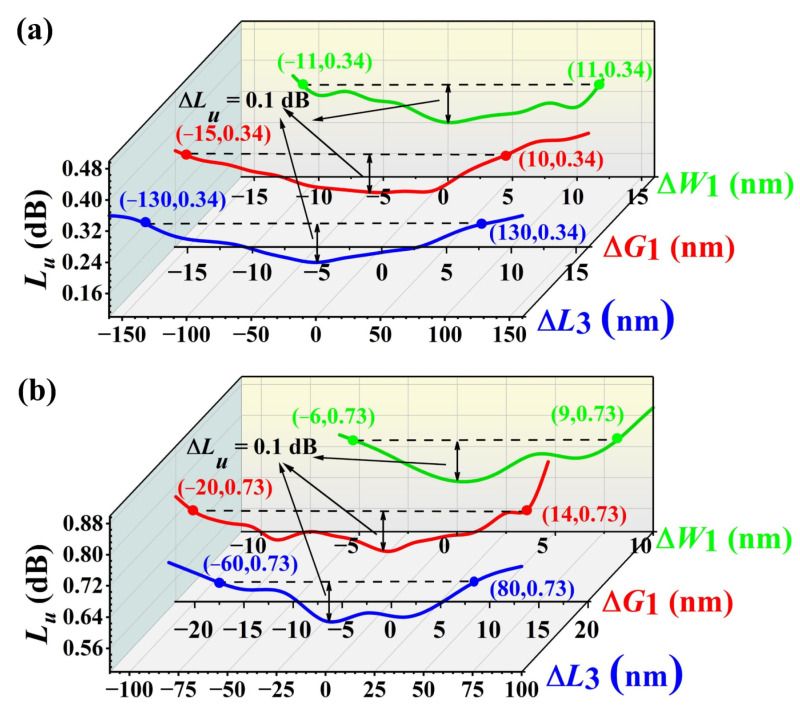
Simulated non-uniformity for (**a**) 8 and (**b**) 16-channel nanowire-assisted AWG with the variation of Δ*W*_1_, Δ*G*_1_ and Δ*L*_3_.

It is crucial to perform a sensitivity analysis of the device and demonstrate its robust. For the sensitivity analysis, the above constraint for parameters optimization should also be met. As shown in Figure 7, the sensitivities of 8- and 16-channel AWGs were simulated. Variations in non-uniformity were simulated by applying offsets to the AWG parameters *W*_1_, *G*_2_, and *L*_3_. For a 8-channel AWG, when Δ*W*_1_ is between −11 and 11 nm, Δ*G*_1_ is varying from −15 to 10 nm, and Δ*L*_3_ is between −130 and 130 nm, the non-uniformity lies in the range from 0.24 to 0.34 dB, as shown in Figure 7a. For a 16-channel AWG, when Δ*W*_1_ is between −6 and 9 nm, Δ*G*_1_ is varying from −20 to 14 nm, and Δ*L*_3_ is between −60 and 80 nm, the non-uniformity lies in the range from 0.63 to 0.73 dB, as shown in Figure 7b. Thus, for the 8-channel AWG, with the fabrication tolerance for *W*_1_, *G*_2_, and *L*_3_ of 22, 25, and 260 nm, respectively, the maximum variation of non-uniformity is 0.1 dB. For the 16-channel AWG, with the fabrication tolerance for *W*_1_, *G*_2_, and *L*_3_ of 15, 34, and 140 nm, respectively, the same non-uniformity change can be obtained. It should be mentioned that some phase noise would be introduced during the lithography. When the phase noise increases from 0 to 1 rad, the simulated noise floor of the 8- and 16-channel AWG would increase 4.16 and 6.14 dB, respectively. Thus, an optimized fabrication process is crucial for the device production.

We also compare the presented design with other reported results as in Table 2. The proposed AWG can perform better in improving the non-uniformity without introducing any additional insertion loss. At the same time, the waveguide size enables its fabrication by commercially available manufacturing facilities, which could facilitate its low-cost applications. The proposal of this scheme is quite simple for improving AWG performance, which is expected to be applied in other multi-parameter uniformity optimization.

## 4. Conclusions

In summary, a silicon nanowire-assisted AWG is proposed, which can achieve a high uniformity with a low insertion loss. In comparison with conventional 8- and 16-channel AWGs for channel spacing of 200 GHz, the non-uniformity of the presented structure can be improved from 1.09 and 1.6 dB to 0.24 and 0.63 dB, respectively. The overall footprint of the device could remain identical, which is 276 × 299 or 258 × 303 μm^2^ for the 8 or 16-channel AWG. Moreover, the proposed AWG has the advantages of moderate wire size, which can be fabricated by a commercial CMOS foundry in high volumes at a low cost. The present nanowire-assisted highly uniform silicon-based AWG is of great significance for the development of integrated DWDM systems.

## Figures and Tables

**Table 1:** Parameters of the nanowire-assisted AWG.

Design Parameter		
Number of channels	8	16
Center wavelength (nm)	1556	1556
Channel spacing (nm)	1.6	1.6
Free spectral range (nm)	23.39	25.99
Single mode waveguide width (nm)	500	500
Diffraction order	40	36
Length increment (μm)	25.34	22.80
Pitch of adjacent arrayed waveguides (μm)	1.4	1.4
Length of star coupler (μm)	30	50
Number of arrayed waveguides	26	24
Number of nanowires	2	2
Spacing between nanowires and arrayed waveguides (nm)	165	165
Width of nanowires (nm)	230	230
Length of nanowires (μm)	3.5	3.5

**Table 2:** Comparison of different high uniformity AWGs reported recently.

Structures	Non-Uniformity	Channels	Additional Insertion Loss	Cross-Talk	Year
Conventional [8]	3	15	3.5 dB	−19 dB	2017
Optical Combiner Structures [30]	1.8	32	4.65 dB	−38 dB	2009
AWG with MMI [33]	0.8 dB	12	2.07 dB	−19.5 dB	2013
Parabolic MMI [39]	1.4 dB	10	2 dB	−25.4 dB	2015
Dual-tapered assisted waveguides [21]	1.9 dB	15	1.1 dB	−15 dB	2018
Cyclic Arrayed waveguides [22]	1.02 dB	16	2.45 dB	22 dB	2019
Mode field converters [31]	0.5 dB	16	1.524 dB	−32 dB	2019
This work	0.24/0.63 dB	8/16	0 dB	−27/−20.7 dB	2022

## Data Availability

The data that support the findings of this study have not been made available but can be obtained from the author upon request.

## References

[1] Liu M., Yin X.B., Ulin-Avila E., Geng B.S., Zentgraf T., Ju L., Wang F., Zhang X. (2011). A graphene-based broadband optical modulator. Nature.

[2] Li M.X., Huang Z.N., Liu Z.Y., Jiang C., Mou C.B., Liu Y.Q. (2021). Tunable Broadband Mode Converter Based on Long-Period Fiber Gratings at 2-μm Waveband. J. Light. Technol..

[3] Blau M., Marom D.M. (2015). Spatial aperture-sampled mode multiplexer extended to higher mode count fibers. J. Light. Technol..

[4] Chen Y., Tang W. (2010). Reconfigurable asymmetric optical burst switching for concurrent dwdm multimode switching: Architecture and research directions [topics in optical communications]. IEEE Commun. Mag..

[5] Hao Y.L., Wu Y.M., Yang J.Y., Jiang X.Q., Wang M.H. (2006). Novel dispersive and focusing device configuration based on curved waveguide grating (CWG). Opt. Express.

[6] Ma X., Yu J., Hua X., Wei C., Huang Y., Yang L., Li D., Hao Q., Liu P., Jiang X. (2014). LioeSim: A network simulator for hybrid opto-electronic networks-on-chip analysis. J. Light. Technol..

[7] Ye T., Fu Y., Qiao L., Chu T. (2014). Low-crosstalk Si arrayed waveguide grating with parabolic tapers. Opt. Express.

[8] Zou J., Le Z.C., Hu J.H., He J.J. (2017). Performance improvement for silicon-based arrayed waveguide grating router. Opt. Express.

[9] Okamoto K., Ishida K. (2013). Fabrication of silicon reflection-type arrayed-waveguide gratings with distributed Bragg reflectors. Opt. Lett..

[10] Doerr C.R., Zhang L., Winzer P.J. (2010). Monolithic InP multiwavelength coherent receiver using a chirped arrayed waveguide grating. J. Light. Technol..

[11] Takada K., Yamada H., Inoue Y. (1996). Optical low coherence method for characterizing silica-based arrayed-waveguide grating multiplexers. J. Light. Technol..

[12] Lu S., Yang C.X., Yan Y.B., Jin G.F., Zhou Z.Y., Wong W.H., Pun E. (2005). Design and fabrication of a polymeric flat focal field arrayed waveguide grating. Opt. Express.

[13] Wang J., Sheng Z., Li L., Pang A., Wu A., Li W., Wang X., Zou S., Qi M., Gan F. (2014). Low-loss and low-crosstalk 8 × 8 silicon nanowire AWG routers fabricated with CMOS technology. Opt. Express.

[14] Park J., Joo J., Kwack M.J., Kim G., Han S.P., Kim S. (2021). Three-dimensional wavelength-division multiplexing interconnects based on a low-loss SixNy arrayed waveguide grating. Opt. Express.

[15] Soref R. (2006). The past, present, and future of silicon photonics. IEEE J. Sel. Top. Quantum Electron..

[16] Bogaerts W., Selvaraja S.K., Dumon P., Brouckaert J., De Vos K., Van Thourhout D., Baets R. (2010). Silicon-on-insulator spectral filters fabricated with CMOS technology. IEEE J. Sel. Top. Quantum Electron..

[17] Won R. (2010). Integrating silicon photonics. Nat. Photonics.

[18] Atabaki A.H., Eftekhar A.A., Askari M., Adibi A. (2013). Accurate post-fabrication trimming of ultra-compact resonators on silicon. Opt. Express.

[19] Liu H., Feng J., Ge J., Zhuang S., Yuan S., Chen Y., Li X., Tan Q., Yu Q., Zeng H. (2021). Tilted Nano-Grating Based Ultra-Compact Broadband Polarizing Beam Splitter for Silicon Photonics. Nanomaterials.

[20] Nishi H., Tsuchizawa T., Kou R., Shinojima H., Yamada T., Kimura H., Ishikawa Y., Wada K., Yamada K. (2012). Monolithic integration of a silica AWG and Ge photodiodes on Si photonic platform for one-chip WDM receiver. Opt. Express.

[21] Song G.Y., Wang S.X., Zou J., Lang T.T., He J.J. (2018). Silicon-based cyclic arrayed waveguide grating routers with improved loss uniformity. Opt. Commun..

[22] Xia X., Lang T.T., Zhang L.B., Yu Z.H. (2019). Reduction of non-uniformity for a 16 × 16 arrayed waveguide grating router based on silica waveguides. Appl. Opt..

[23] Piels M., Bauters J.F., Davenport M.L., Heck M.J.R., Bowers J.E. (2014). Low-Loss Silicon Nitride AWG Demultiplexer Heterogeneously Integrated With Hybrid III–V/Silicon Photodetectors. J. Light. Technol..

[24] Yu R., Cheung S., Li Y., Okamoto K., Proietti R., Yin Y., Yoo S.J.B. (2013). A scalable silicon photonic chip-scale optical switch for high performance computing systems. Opt. Express.

[25] Alexoudi T., Terzenidis N., Pitris S., Moralis-Pegios M., Maniotis P., Vagionas C., Mitsolidou C., Mourgias-Alexandris G., Kanellos G.T., Miliou A. (2019). Optics in computing: From photonic network-on-chip to chip-to-chip interconnects and disintegrated architectures. J. Light. Technol..

[26] Lee J.H., Choi H.Y., Shin S.K., Chung Y.C. (2006). A Review of the Polarization-Nulling Technique for Monitoring Optical-Signal-to-Noise Ratio in Dynamic WDM Networks. J. Light. Technol..

[27] Sakamaki Y., Kamei S., Hashimoto T., Kitoh T., Takahashi H. (2009). Loss uniformity improvement of arrayed-waveguide grating with mode-field converters designed by wavefront matching method. J. Light. Technol..

[28] Hashimoto T., Saida T., Ogawa I., Kohtoku M., Shibata T., Takahashi H. (2005). Optical circuit design based on a wavefront-matching method. Opt. Lett..

[29] Sakamaki Y., Saida T., Hashimoto T., Takahashi H. (2007). New optical waveguide design based on wavefront matching method. J. Light. Technol..

[30] Takiguchi K., Okamoto K., Sugita A. (2006). Arrayed-waveguide grating with uniform loss properties over the entire range of wavelength channels. Opt. Lett..

[31] Chen Y., Wang S.X., Lang T.T., He J.J. (2019). Uniform-loss cyclic arrayed waveguide grating router using a mode-field converter based on a slab coupler and auxiliary waveguides. Opt. Lett..

[32] Kamei S., Ishii M., Kaneko A., Shibata T., Itoh M. (2009). *N* × *N* Cyclic-Frequency Router With Improved Performance Based on Arrayed-Waveguide Grating. J. Lightwave Technol..

[33] Pathak S., Vanslembrouck M., Dumon P., Van Thourhout D., Bogaerts W. (2013). Optimized silicon AWG with flattened spectral response using an MMI aperture. J. Light. Technol..

[34] Sheng Z., Dai D., He S. (2007). Improve channel uniformity of an Si-nanowire AWG demultiplexer by using dual-tapered auxiliary waveguides. J. Light. Technol..

[35] Smit M.K., Van Dam C. (1996). PHASAR-based WDM-devices: Principles, design and applications. IEEE J. Sel. Top. Quantum Electron..

[36] Zou J., Ma X., Xia X., Hu J., Wang C., Zhang M., Lang T., He J.-J. (2020). High resolution and ultra-compact on-chip spectrometer using bidirectional edge-input arrayed waveguide grating. J. Light. Technol..

[37] Chen Y.S., Feng J.J., Chen J., Liu H.P., Yuan S., Guo S., Yu Q.H., Zeng H.P. (2022). Optical Bistability in a Tunable Gourd-Shaped Silicon Ring Resonator. Nanomaterials.

[38] Xiao S., Khan M.H., Shen H., Qi M. (2007). Multiple-channel silicon micro-resonator based filters for WDM applications. Opt. Express.

[39] Pan P., An J., Zhang J., Wang Y., Wang H., Wang L., Yin X., Wu Y., Li J., Han Q. (2015). Flat-top AWG based on InP deep ridge waveguide. Opt. Commun..

